# Bacteriophage ICP1: A Persistent Predator of *Vibrio cholerae*

**DOI:** 10.1146/annurev-virology-091919-072020

**Published:** 2021-07-27

**Authors:** Caroline M. Boyd, Angus Angermeyer, Stephanie G. Hays, Zachary K. Barth, Kishen M. Patel, Kimberley D. Seed

**Affiliations:** 1 Department of Plant and Microbial Biology, University of California, Berkeley, California 94720, USA;; 2 Chan Zuckerberg Biohub, San Francisco, California 94158, USA

**Keywords:** bacteriophage, cholera, genome evolution, recombination, CRISPR-Cas, coevolution

## Abstract

Bacteriophages or phages—viruses of bacteria—are abundant and considered to be highly diverse. Interestingly, a particular group of lytic *Vibrio cholerae*–specific phages (vibriophages) of the International Centre for Diarrheal Disease Research, Bangladesh cholera phage 1 (ICP1) lineage show high levels of genome conservation over large spans of time and geography, despite a constant coevolutionary arms race with their host. From a collection of 67 sequenced ICP1 isolates, mostly from clinical samples, we find these phages have mosaic genomes consisting of large, conserved modules disrupted by variable sequences that likely evolve mostly through mobile endonuclease-mediated recombination during coinfection. Several variable regions have been associated with adaptations against antiphage elements in *V. cholerae*; notably, this includes ICP1’s CRISPR-Cas system. The ongoing association of ICP1 and *V. cholerae* in cholera-endemic regions makes this system a rich source for discovery of novel defense and counterdefense strategies in bacteria-phage conflicts in nature.

## 1. INTRODUCTION

**Figure 1:**
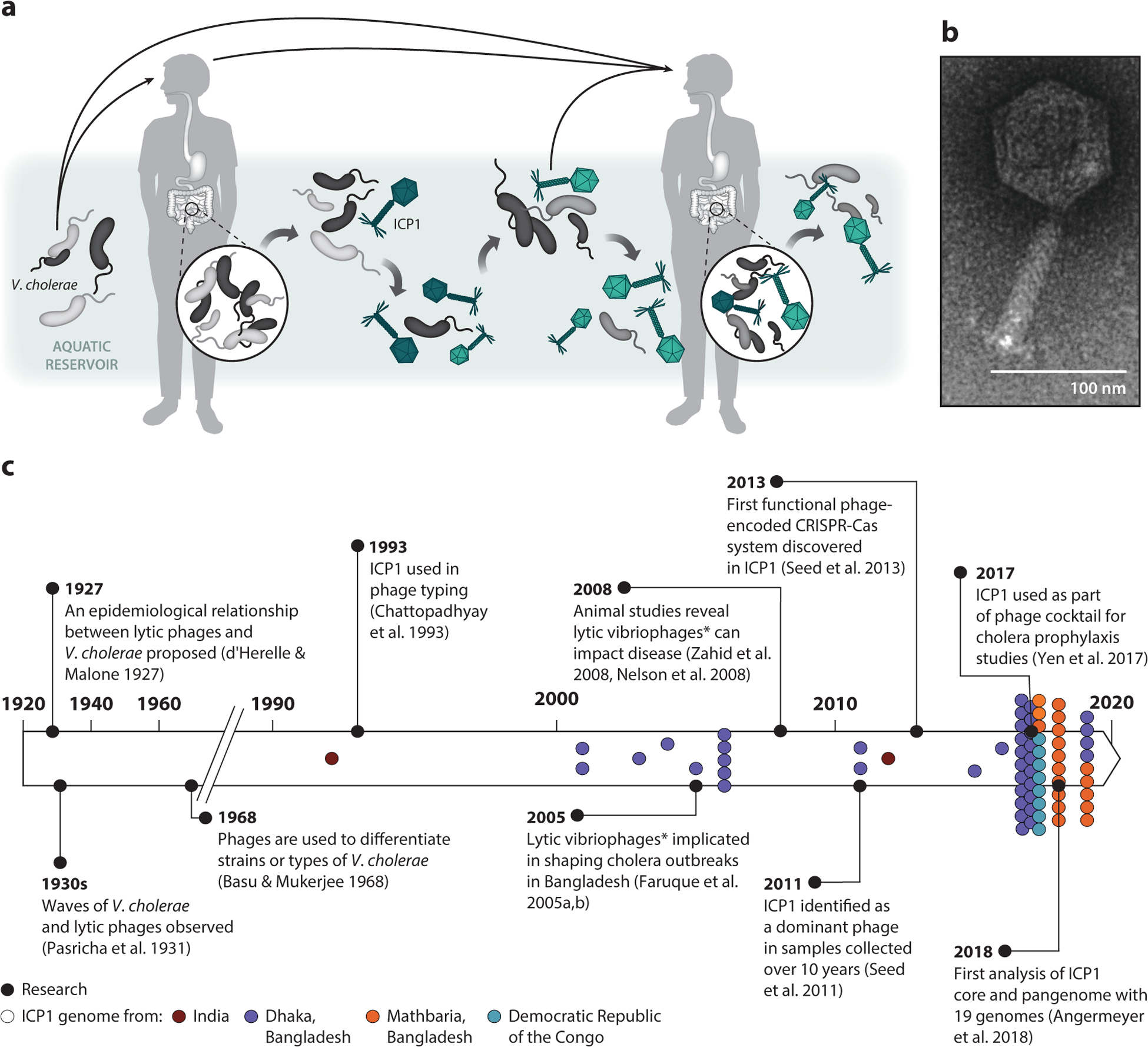
International Centre for Diarrheal Disease Research, Bangladesh cholera phage 1 (ICP1) and epidemic *Vibrio cholerae* coexist and persist in nature. (*a*) Upon ingestion, toxigenic *V. cholerae* strains belonging to the O1 serogroup colonize the gut, causing the diarrheal disease cholera. During disease, *V. cholerae* replicates and is shed in stool, resulting in reseeding of the aquatic environment. The lytic bacteriophage ICP1 (*dark teal*) preys on *V. cholerae* (*light gray*), leading to the selection of phage-resistant strains (*black*). Counteradaptations in the phage lead to selection of new ICP1 variants (*light teal*). This reciprocal coevolution continues (emergence of *dark gray* bacteria) in the aquatic and intestinal environments. How phage predation may influence disease outcome has yet to be determined. (*b*) A transmission electron micrograph of ICP1 (scale bar indicates 100 nm). (*c*) A brief history of the interest in the role of lytic phages in cholera epidemiology and ICP1 research, highlighting notable discoveries and documentation of sequenced isolates, color-coded according to region of isolation. Asterisks indicate that some of the vibriophages used in these studies were later found to be ICP1. Notably, JSF4 used in References 4 and 76 is ICP1_2001_Dha_D.

As a waterborne pathogen and causative agent of the infectious diarrheal disease cholera, *Vibrio cholerae* must persist in aquatic reservoirs and flourish in the human gut. In both environments, *V. cholerae* is attacked by predatory phages, an observation that was first made just under a century ago (1). Unlike lysogenic phages, such as CTX [which encodes the instigator of diarrhea—cholera toxin (2)], lytic phages are uniquely poised to alter bacterial population dynamics as their successful infections result in bacterial death and release of tens to hundreds of phage progeny. A 1927 report correlating cholera patient disease outcome with the co-occurrence of “strong or weak” phage hypothesized that the course of disease is governed by the behavior of lytic phages that prey on *V. cholerae* (1). Seventy-five years later, phage predation was implicated in naturally controlling the duration and severity of cholera epidemics (3, 4). It is thought that in aquatic reservoirs, evolution of resistance and counter-resistance gives rise to cyclical patterns of increasing *V. cholerae* populations, followed by phage amplification and coincident bacterial decline, leading to phage decline and bacterial blooms once again (Figure 1a). These collective observations, alongside the use of phages to identify types of *V. cholerae* to address the clonality of cholera outbreaks (5) and renewed interests in harnessing phages for preventing cholera outbreaks (6), have fostered a long-standing interest in cholera-associated phages. This review focuses on one such lytic phage, International Centre for Diarrheal Disease Research, Bangladesh cholera phage 1 (ICP1), which has emerged as a persistent and pervasive phage shaping the evolution of epidemic *V. cholerae* (Figure 1).

**ICP1:**

International Centre for Diarrheal Disease Research, Bangladesh cholera phage 1

Within the human intestine, robust replication of toxigenic *V. cholerae* results in shedding of the pathogen in stool. This reseeds the aquatic environment and leads to transmission of disease from person to person via fecal-oral contamination and contributes to the explosive nature of cholera outbreaks (7). Although several hundred serogroups of *V. cholerae* with distinct O antigen components of the surface-exposed lipopolysaccharide (LPS) have been described, epidemic cholera is overwhelmingly associated with the O1 serogroup (7). Notably, for O1 *V. cholerae*, O1 is necessary for intestinal colonization (8, 9) and serves as the receptor for the lytic bacteriophage ICP1 (10). ICP1 was first recognized as a dominant phage in cholera patient stool samples collected between 2001 and 2010 in Bangladesh (10). For a span of more than 20 years, ICP1 has consistently been coisolated with *V. cholerae* in this region (11–15). Although whole-genome sequencing has now become routine and the genomes of numerous ICP1 isolates have been published, inconsistent approaches in both naming and annotating phages continue to obscure ICP1’s dominance. In an effort to reconcile this, several previously described vibriophages have been sequenced or reannotated and are now recognized as ICP1 phages (Supplemental Table 1). This includes the oldest known isolate, originally named M4, recovered from sewage in India and published in 1993 (16). Now, it is increasingly evident that ICP1 is a persistent lytic phage associated with epidemic *V. cholerae*. Importantly, the intimate association between ICP1 and epidemic *V. cholerae* that persists in Bangladesh also extends to other geographical locations, including India (12), the Democratic Republic of the Congo (M.T. Alam, T. Pisiae, K.D. Seed, J.G. Morris, Jr., A. Ali, manuscript in preparation), and South Sudan (17). Despite the breadth of time and space between some isolates, and counter to the general expectation regarding the enormous diversity of phages on the planet (18), ICP1 isolates show high nucleotide conservation across their relatively large (~126 kb) double-stranded DNA (dsDNA) genomes. However, slight variations between ICP1 isolates have brought to light novel adaptations enabling ICP1 to persist alongside its ever-changing host, the most notable of which is the acquisition of a fully functional CRISPR-Cas system targeting an antiphage element in *V. cholerae*’s genome (11). The reciprocal adaptations that allow for a balance between ICP1 and *V. cholerae* populations hold a wealth of information about coevolutionary arms races in nature. Tools developed for genetic manipulation of ICP1 (19, 20) and the application of systems-level -omics approaches (21, 22) provide a unique opportunity to gain a mechanistic understanding of this phage and its effect on epidemic *V. cholerae*. In this review, we summarize the current knowledge of ICP1’s biology, features of its genome, and its relationship to other known phages to address how each contribute to ICP1’s persistent coexistence with *V. cholerae*.

**LPS:**

lipopolysaccharide

**Vibriophage:**

bacteriophage that infects *Vibrio* species

**CRISPR-Cas:**

clustered regularly interspersed short palindromic repeats-CRISPR-associated proteins

## 2. THE ICP1 LIFE CYCLE

**Figure 2:**
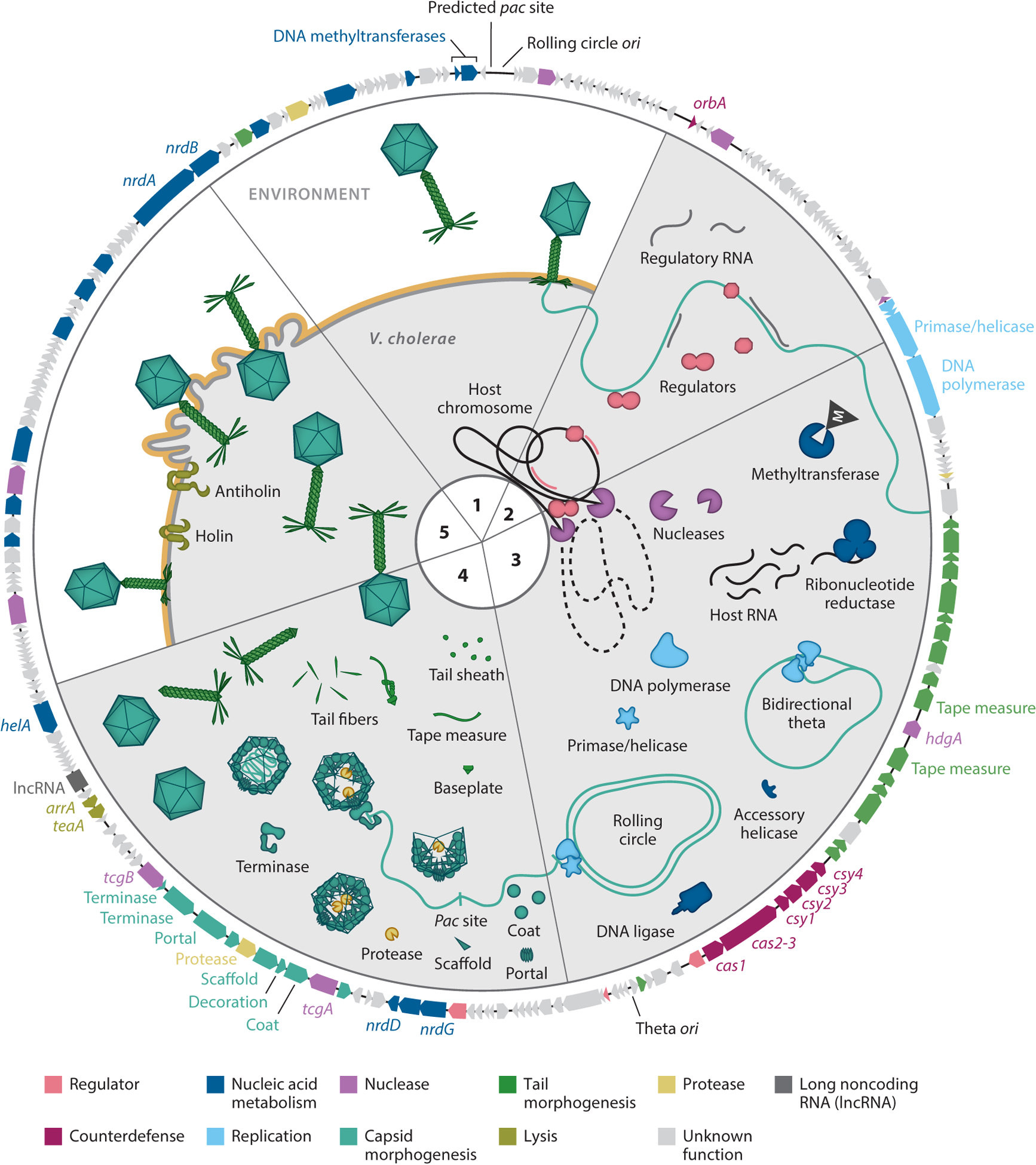
Overview of the International Centre for Diarrheal Disease Research, Bangladesh cholera phage 1 (ICP1) lytic life cycle. ICP1’s life cycle can be roughly divided into five phases. The outer ring is a representative ICP1 genome (2006_Dha_E). Genes and proteins are colored according to function. Phase 1: ICP1 binds to the O1 antigen (*yellow*) (10) of *Vibrio cholerae* (*light gray*) and injects its genome (*light teal*). Phase 2: ICP1 initiates a temporally controlled gene expression cascade that includes the expression of regulators and abundant noncoding RNAs (21). Phase 3: ICP1 uses its nucleases to liberate nucleotides (14) and nucleic acid metabolism genes to modify host nucleic acids for replication of its genome, first via bidirectional theta and then by rolling circle replication (21), using the latter to produce concatemerized genomes; the different origins of replication (*ori*) and approximate packaging recognition (*pac*) site are indicated on the genome. Phase 4: ICP1 synthesizes tails and procapsids, packages replicated genomes using headful packaging, and then assembles virions. Phase 5: ICP1 regulates the release of progeny via holin- and antiholin-mediated lysis (41). Under laboratory conditions, ICP1 produces ~90 infectious progeny within 20 min of infection (23).

Lytic phages infect susceptible bacteria and commandeer cellular metabolism to promote phage replication, ultimately producing a large number of infectious phage particles that are released through cell lysis. While much remains to be discovered about the mechanisms ICP1 uses to produce an impressive burst of approximately 90 progeny virions within 20 minutes (min) of infection (23), certain aspects of ICP1’s life cycle under laboratory conditions have been elucidated. In this section, we describe what is currently understood about ICP1’s life cycle (largely inferred from experiments with ICP1_2006_Dha_E) (Figure 2). Where experimental evidence is incomplete, we rely on homology to infer function. ICP1 isolates have conserved genes for genome replication, nucleic acid metabolism, virion morphogenesis, and lysis; thus, we anticipate that the details that have been worked out for ICP1_2006_Dha_E broadly apply to all phages in the ICP1 lineage. What is harder to predict, however, is how the dynamics of the life cycle are affected by environmental contexts or host genotypes. Future studies examining these variables will provide information about the potential effect of ICP1 on epidemic *V. cholerae* during its residence in both the aquatic reservoir and the human intestinal tract.

### 2.1. Gene Expression and Regulation

Immediately after initiating infection by binding to the O1 antigen of *V. cholerae*’s LPS (10) and injecting its genome, ICP1 reprograms *V. cholerae* for phage reproduction by implementing a tightly regulated gene expression program (22). First, early genes reach peak expression at 4 min postinfection and have largely unknown functions, but as in other phages, they are likely to play important roles in host cell takeover and countering host defenses. For example, OrbA, which counters a *V. cholerae* defense system known as bacteriophage exclusion (BREX), shows peak expression early in infection, consistent with its role in subverting host defense (15). Next, middle genes involved in nucleic acid metabolism and DNA replication dominate the transcriptional landscape. Finally, late genes reach peak expression around 16–20 min postinfection and encode structural proteins needed for virion assembly and proteins involved in cell lysis. As can be expected based on their function, ICP1’s capsid morphogenesis genes are highly expressed and are among the most abundant transcripts in the cell late in infection. Although the temporally coordinated gene expression program for ICP1 could be anticipated, there were several surprises uncovered within the transcriptome (22). Of particular interest, ICP1 abundantly expresses noncoding RNAs (ncRNAs). In fact, one of the most abundantly expressed ICP1-encoded transcripts is predicted to encode a long ncRNA (lncRNA) that is expressed robustly throughout infection. The function of this lncRNA warrants further investigation, especially given its conservation near lysis genes not only in ICP1 phages but also in other related phages (see Section 3.3). Unfortunately, although ICP1’s gene expression patterns are now documented, little is known about how ICP1 controls its expression program. Of note, ICP1 is not predicted to encode its own RNA polymerase, indicating that it must redirect *V. cholerae*’s polymerase. How this is achieved and if there are mechanistic parallels to how the well-studied T4 phage mediates control over *Escherichia coli*’s RNA polymerase (24) remain to be seen.

**BREX:**

bacteriophage exclusion

### 2.2 Genome Replication and Nucleic Acid Metabolism

Viruses hijack host resources. Nucleotides can come from the degradation of the host DNA (25), and there is evidence that ICP1 employs this strategy during infection (14). The liberated nucleotides can be scavenged, and other host nucleic acids can be modified by ICP1’s nucleic acid metabolism proteins to be incorporated into newly synthesized genomes. Like T7 and T5 phages, ICP1 encodes replication machinery consisting of a DNA polymerase I gene product (Gp58) and a GP4-type DNA helicase primase (Gp57) (21). However, the complete replisome for ICP1 has yet to be characterized. ICP1’s replication dynamics under laboratory conditions, in contrast, have been documented and are described in detail in Reference 21. Initially, at about 8 min postinfection, replication of ICP1’s genome occurs via the bidirectional theta mechanism starting from the origin of replication (*ori*) located between the tail and capsid modules. Later, around 12–16 min postinfection, a switch occurs and genomes are synthesized via rolling circle replication from an alternative *ori* near the predicted packaging recognition (*pac*) site (21). While it is not known what controls the switch to rolling circle replication, it is important as this process produces genome concatemers, the necessary substrates for packaging into assembled procapsids.

**Gp:**

gene product

### 2.3. Virion Assembly

Although ICP1 virions have not yet been subjected to complete proteomic analyses, the structural proteins that make up ICP1’s virions with their 84-nm-wide icosahedral capsids and 20 × 100 nm contractile tails (10, 20, 26, 27) (Figure 1b) are readily predicted given their shared motifs across viral families. We have predicted how ICP1 virions are likely assembled from the conservation of the general assembly pathway of dsDNA viruses (28). Most components of the tail are encoded by Gp69–93, which, curiously, are disrupted by the CRISPR-Cas genes. As ICP1 is a myovirus, its tail likely assembles in a manner similar to T4 phage (29). As such, we predict that the baseplate, comprising several subunits (Gp72–78), assembles first. The tail tube (Gp83) is then built from this platform with the aid of a chaperone (Gp82). Tail length is determined by the length of the fully extended tape measure protein (Gp79–81). It is notable that the tape measure gene is interrupted by a mobile endonuclease gene, which we hypothesize gets spliced out at the RNA level. This would result in an ~2,000-bp-long gene, corresponding to ~100 nm in length when assuming about 0.15 nm per amino acid (30), and matches the lengths observed by transmission electron microscopy. The tail is coated by the sheath proteins (Gp84), and capping of the tail likely involves the addition of the head-tail connector or neck protein (Gp86). Finally, tail fibers (Gp69, Gp70, Gp93) are added to the baseplate to form a complete tail, which attaches to a capsid at the neck protein after DNA packaging.

Similar to tail assembly, we can predict ICP1’s capsid assembly pathway from other well-characterized phages (31–37). The procapsid is assembled from coat proteins (Gp122), which are guided by scaffolds (Gp124) into place around the portal (Gp127) through which the concatemerized genomes are packaged by the terminase. The two-subunit terminase recognizes genomes at their *pac* site and translocates the DNA into the procapsid. While the large terminase is typically easy to identify (ICP1’s is Gp129–128), the small terminase is more divergent between viruses and remains to be identified in ICP1. Near-identical sequences for both the large terminase and the *pac* site in all 67 ICP1 isolates suggest the small terminase is likely one of ICP1’s highly conserved proteins. Genome packaging is expected to stimulate scaffold release, presumably by protease-mediated (Gp125) degradation, leading to a conformational change in the capsid that is stabilized by the addition of the decoration protein (Gp123). ICP1 lacks the genomic sequences to start and stop genome packaging as required for *cos* packaging and instead is predicted to use a headful packaging mechanism (21). With this strategy, typically more than 100% of the genome is packaged (38). Considering the diversity in genome sizes we observe in ICP1 isolates, differing by ~7.5%, and the conservation of the structural genes, perhaps this headful packaging strategy is useful for allowing flexibility of genome size as the phage evolves.

### 2.4. Lysis

Like all lytic phages, ICP1 lyses its host to release newly assembled progeny from the confines of the dying cell. Phage-mediated lysis requires breaking through the inner membrane, peptidoglycan, and outer membrane in Gram-negative bacteria, which is typically mediated by a tripartite system comprising a holin, endolysin, and spanin (39, 40). TeaA (Gp137) was experimentally validated as ICP1’s holin (41). Although the endolysin and spanin in this system remain to be characterized, ArrA (Gp138) was found to be an antiholin, which delays lysis (41). Antiholin-mediated lysis inhibition is a strategy ICP1 uses to delay the release of virions based on the abundance of other ICP1 in the surrounding environment. On the one hand, in an environment rich in uninfected hosts, it is advantageous for ICP1 to release its progeny quickly, within 20 min of infection, to rapidly infect nearby cells and increase its population exponentially. On the other hand, when high numbers of phage in the environment lead to superinfection of a cell, ICP1 initiates lysis inhibition through unknown mechanisms and delays lysis for up to 90 min. Not only does this delay benefit ICP1 by allowing for a higher probability of progeny virions finding an uninfected host, but also it allows more time within the host to evolve and assemble virions, leading to a larger number of potentially more fit progeny (41).

## 3. ICP1 PHAGE GENOMICS

### 3.1. Genomic Conservation and Diversity

**Figure 3:**
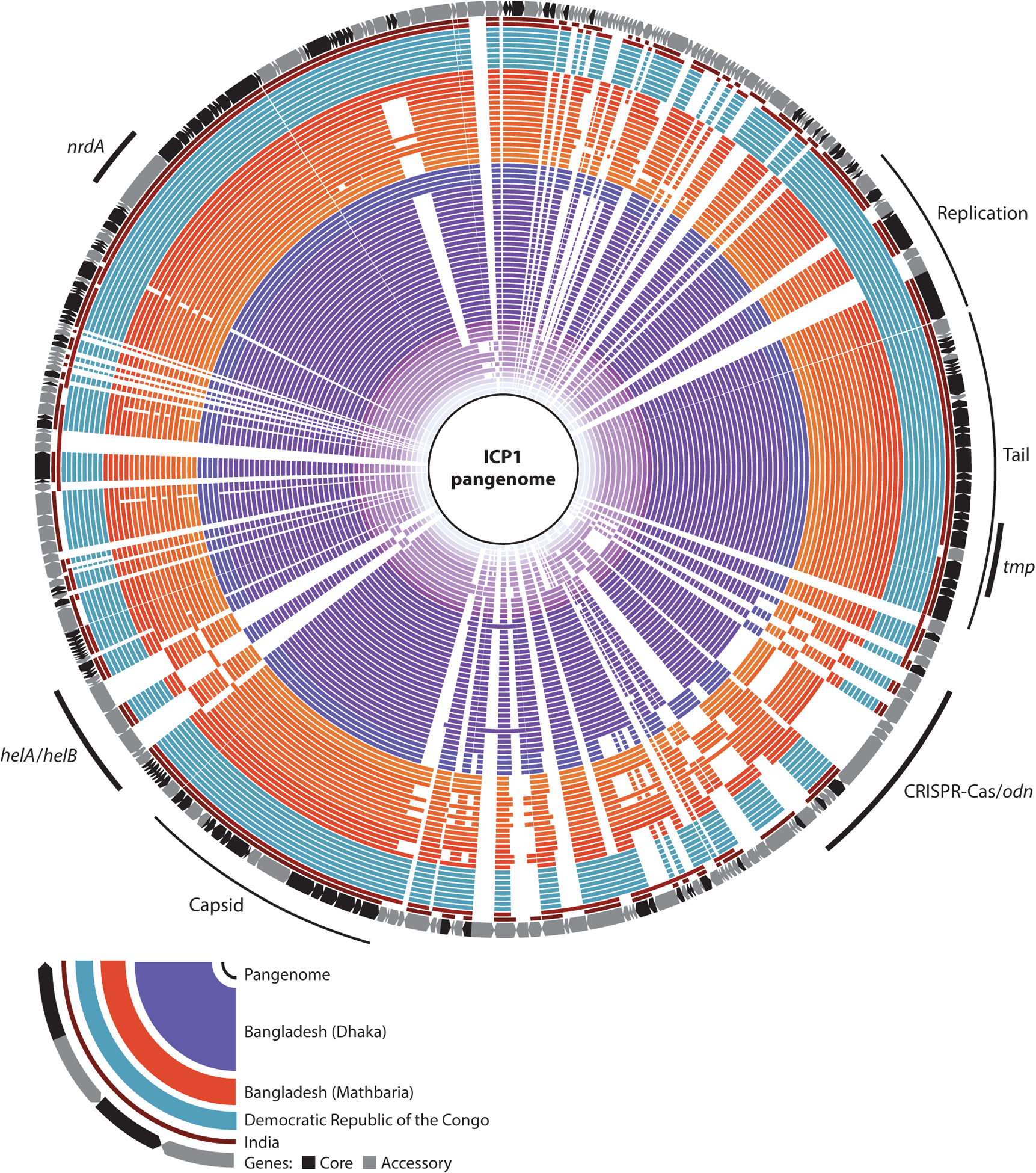
Pangenome of the 67 International Centre for Diarrheal Disease Research, Bangladesh cholera phage 1 (ICP1) isolates. ICP1’s pangenome is highly conserved. The rings are defined in the legend. Within each region, the isolation date is indicated by shading where darker is more recent (complete isolate details are in Supplemental Table 1). Core genes are present in all isolates at greater than or equal to 60% amino acid similarity while accessory genes are absent or nonidentical in one or more isolates. Blank spaces represent the absence of sequence that is found in one or more isolates. Highly conserved modules encoding replication, tail morphogenesis, or capsid morphogenesis genes are indicated by thin lines. Regions with mobile genetic elements (*helA/B*, *nrdA*, *tmp*, CRISPR-Cas*/odn*) are indicated by thick lines in both conserved and variable regions (details of these regions are in Figure 4).

ICP1 is remarkable for its high genetic conservation across both geographic space (Bangladesh, India, the Democratic Republic of the Congo) and a time span of more than 20 years (Figure 3). In our collection of 67 isolates, there is an average of 99.3% (SD 0.49%) nucleotide conservation over 92.6% (SD 5.46%) of each genome’s length (which varies from 121 to 131 kb, for an average of 126 kb), making these phages, to the best of our knowledge, one of the most conserved lineages yet discovered. Between the most distantly related isolates, temporally separated by over 25 years, there is still 98.46% nucleotide identity over 78.48% of the genome, and some isolates recovered several years apart have 99.67% identity over 100% of the genome. An overview of the comparison between all 67 isolates reveals large contiguous regions of core genes that are interspersed with regions of variability where open reading frames (ORFs) or entire operons are swapped out for alternatives (Figure 3). Variable regions appear to be associated with emergence or loss of one or more genes, exchange of genes involved in counterdefense (42), or functionally redundant alleles (14). At the protein level, we predict isolates encode between 217 and 231 ORFs. In the pangenome, there are 340 ORFs of which 144 (~42%) are core genes (i.e., present in all genomes) and 196 comprise the accessory genome of which only 47 are singletons (Supplemental Table 2). This large core genome helps explain much of the genomic conservation of ICP1, while the accessory components hint at a functional modularity that likely allows ICP1 to stay competitive in its coevolutionary arms race with *V. cholerae*. One region of hypervariability (top, Figure 3) that distinguishes isolates from Asia and the Democratic Republic of the Congo lies within the early expressed genes predicted to be involved in host cell takeover, perhaps suggesting selection for alleles able to overcome genetically distinct *V. cholerae* variants in different geographic locations. Notably, a recent study of glacial microcosms revealed that viral diversity remains low between isolates in that environment as well (43). Together, these data may suggest that viral sequence conservation over time is not as rare as previously thought, but rather that some viruses, such as those infecting highly adapted species, retain large core genomes.

**ORF:**

open reading frame

**Pangenome:**

the complete set of genes found in all isolates of a lineage

### 3.2. Recombination and Mobile Genetic Elements

**Figure 4:**
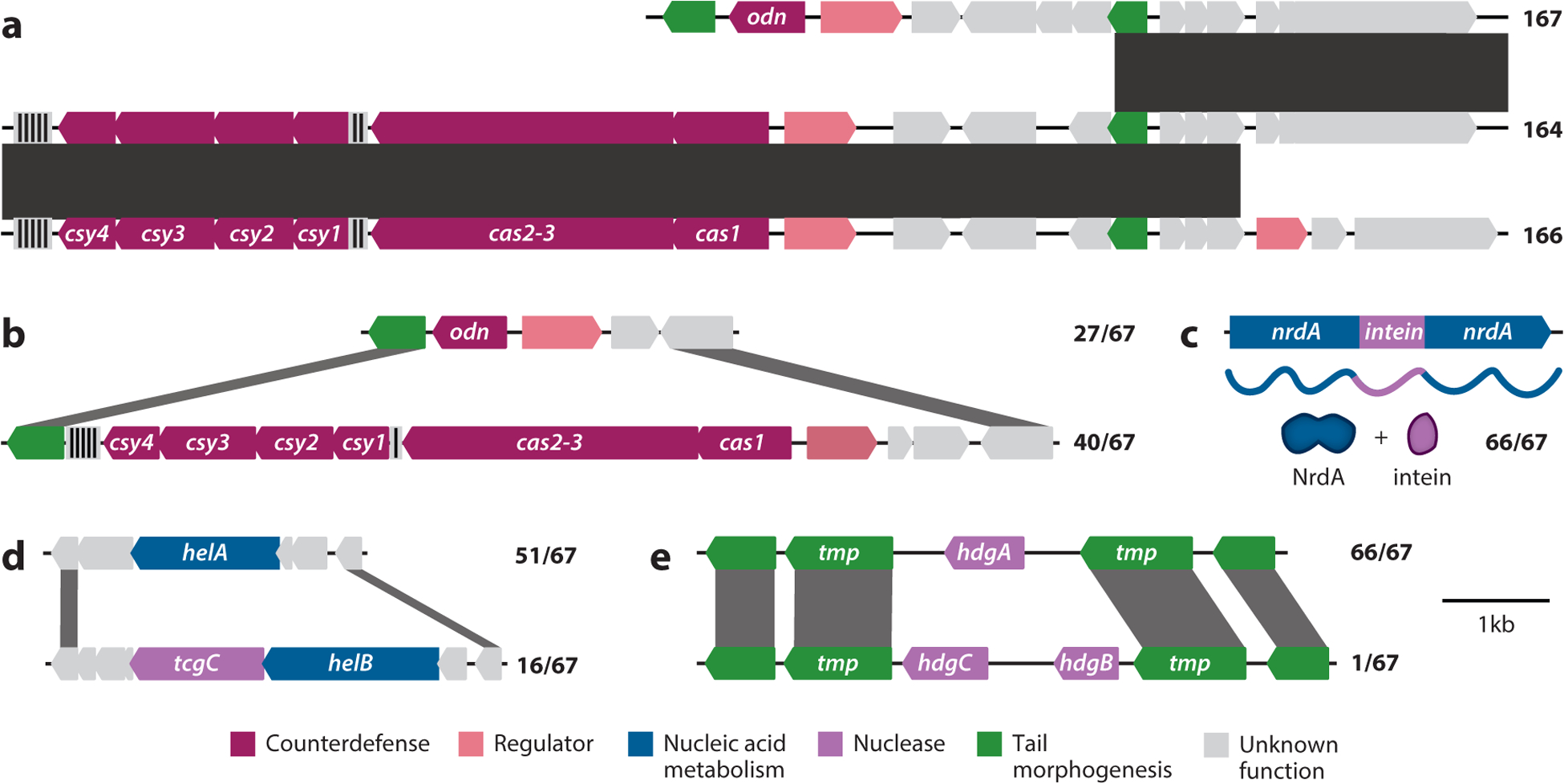
Recombination-driven evolution in International Centre for Diarrheal Disease Research, Bangladesh cholera phage 1 (ICP1): representative isolates and comparisons of a subset of mobile genetic elements (MGEs) and their genomic context within ICP1. These variable loci are flanked by conserved sequences (protein identity greater than 97% for *panels b*, *d*, and *e*; source data are in Supplemental Table 3). Color-coded functions match those from Figure 2, and ratios denote how many of the 67 isolates have each allele. (*a*) Three intrapatient isolates of ICP1 (2018_Mat_167, 2018_Mat_164, and 2018_Mat_166) illustrate recombination near a counterdefense locus, indicated by gray bars showing 100% nucleotide identity between genomes. The vertical black and gray lines on the sequence indicate CRISPR loci, where the number of spacers is indicated by the black lines. (*b*) An ICP1-encoded counterdefense locus is occupied by one of two nucleases, *odn* or *cas2*-*3* of the CRISPR-Cas system. (*c*) The putative intein is schematized showing its presence in the DNA sequence, translated peptide, and absence in the mature NrdA protein. (*d*) One allele of the accessory helicase, *helA*, lacks a neighboring nuclease while *helB* is next to a T5orf172 domain coding gene, *tcgC*. (*e*) Most ICP1 isolates have one HNH domain coding gene, *hdgA*, which splits the tape measure (*tmp*) into two genes, while one isolate (1992_Ind_M4) has two HNH domain coding genes, *hdgB* and *hdgC* in this locus.

Given the high degree of nucleotide conservation between ICP1 isolates, recombination during coinfection is expected to be a major driving force behind ICP1’s evolution. Such recombination is evident not only from laboratory experiments (41) but also more strikingly from cholera patient stool where sequencing of multiple ICP1 isolates from a single patient reveals novel recombinant genotypes (Figure 4a). Recombination is likely accelerated by the high density of endonuclease genes within ICP1’s genome. While it is impossible to conclude what these genes do through sequence alone, for many, sequence comparisons and syntenic context suggest that they are homing endonuclease genes (HEGs), a class of selfish mobile genetic elements (MGEs). A HEG encodes an endonuclease that cleaves specific sequences found in cognate HEG-less loci. Sequence cleavage is followed by recombinational repair of the locus using the uncleaved, HEG-encoding locus as a template. In conditions of heterozygosity, such as coinfection by two distinct viruses, HEGs are able to home into unoccupied sites and drive their inheritance among newly synthesized DNA (44).

**HEG:**

homing endonuclease gene

**MGE:**

mobile genetic element

**Figure 5:**
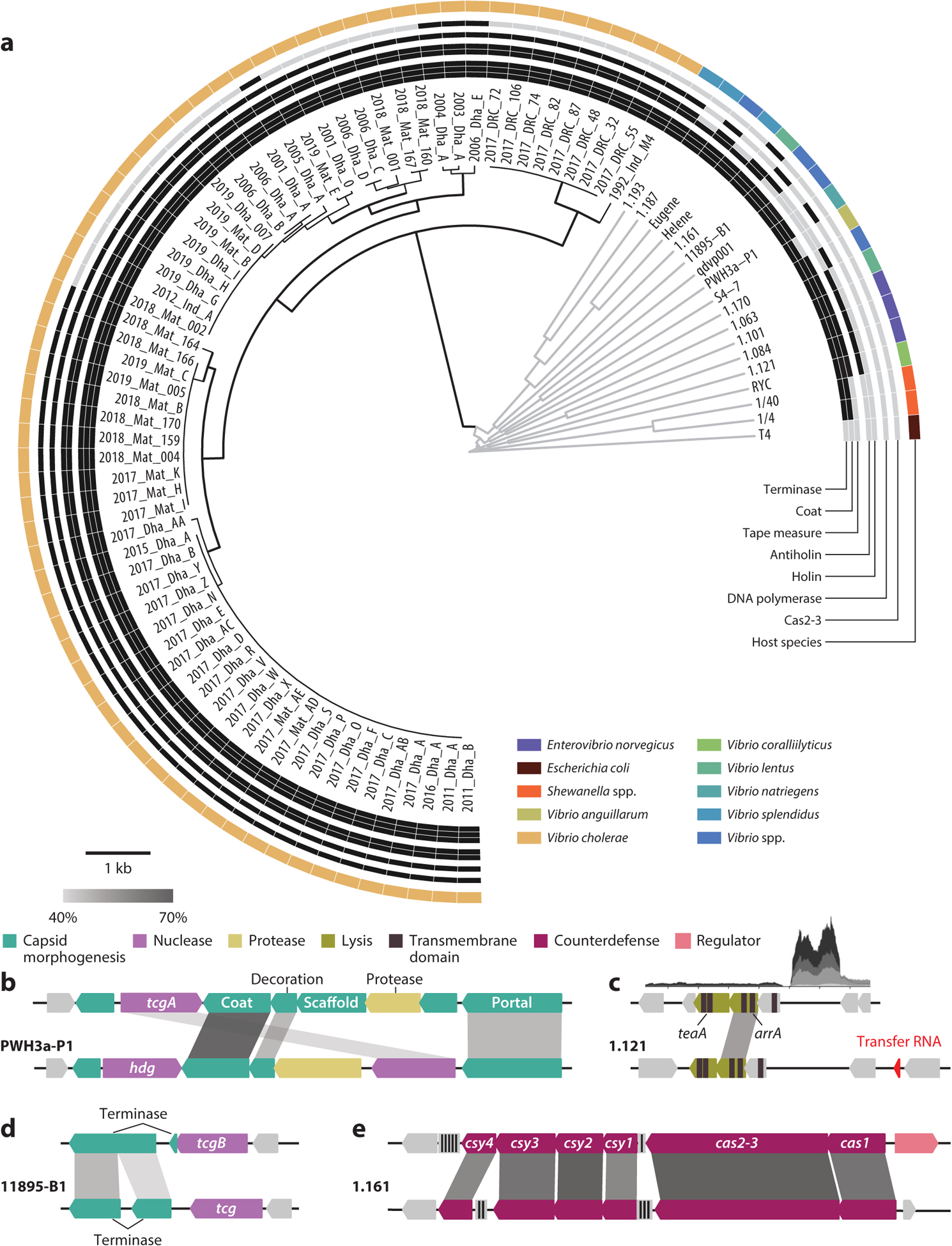
International Centre for Diarrheal Disease Research, Bangladesh cholera phage 1 (ICP1) isolates form a distinct lineage but share notable features with phages infecting other species. (*a*) Phylogenetic tree of the 67 ICP1 isolates (*black branches*) and related phages (*gray branches*), with T4 included as an outgroup, comparing all translated open reading frames to determine overall genome similarity [based on the tBLASTx algorithm from ViPTree (51)]. Outer rings show presence (*black*) or absence (*gray*) of homologs to ICP1-encoded proteins, defined as sharing at least 45% protein similarity over 85% of the sequence. The final ring shows the phage’s bacterial host. Each ICP1 isolate is named based on the year and location of isolation (see Supplemental Table 1 for additional information), and some phage names are shortened for space (see Supplemental Table 4 for additional information). For panels *b*–*e*, protein identity is indicated by gray shading according to the scale (source data are in Supplemental Table 5), and gene function is color coded as in Figure 2. Genetic comparison is shown between (*b*) the capsid operon from ICP1 (*top*) and phage PWH3a-P1, (*c*) the lysis locus of ICP1 (*top*) and phage 1.121 (the inset shows transcriptomic data of the long noncoding RNA in ICP1 at 0, 4, 8, 12, and 16 min postinfection) (22), (*d*) large terminase-encoding genes from ICP1 (*top*) and phage 11895-B1, and (*e*) the CRISPR-Cas locus in ICP1 (*top*) and phage 1.161. The vertical black and gray lines on the sequence indicate CRISPR loci, where the number of spacers is indicated by the black lines.

Although originally found in self-splicing introns within the endosymbiont genomes of certain eukaryotes, the majority of HEGs are thought to be encoded as free-standing genes within phage genomes (44, 45). HEG occurrence has been extensively documented in T4-like phages (46), which appear to have more HEGs than most phage genomes. While T4’s ~160-kb genome has 15 HEGs, ICP1’s ~40-kb smaller genome has ~9 putative HEGs. Despite superficial similarities (both are members of the *Myoviridae* family), most of the core ICP1 and T4 modules lack notable similarity (Figure 5a). However, some auxiliary genes may have shared mechanisms in ICP1 and T4. For example, expression of the T4 accessory helicase Dda was able to phenocopy ICP1’s SF1B-type helicases, HelA or HelB, in deletion strains (14). Although the specific in vivo functions of Dda, HelA, and HelB have not been worked out, Dda has been implicated in replication, repair, and recombination pathways in T4 (47), the same functions that facilitate endonuclease homing. Such shared pathways in ICP1 and T4 might contribute to making them good hosts for homing elements.

Within ICP1, the majority of putative HEGs contain a T5orf172 domain. The T5orf172 domain is a member of the GlyIleTyr-TyrIleGly (GIY-YIG) superfamily of endonuclease domains, but few proteins with this domain have been biochemically characterized. A notable exception is Odn, an ICP1 protein involved in counterdefense (42). This counterdefense locus is occupied by either CRISPR-Cas or *odn* in different ICP1 isolates and is flanked by homologous sequence that allows for recombination (Figure 4b), perhaps driven by the catalytic activity of each locus’s nuclease (Odn or Cas2-3) (42). The sequence outside of the T5orf172 domain in *odn* is more divergent than those in the other ICP1 T5orf172 domain coding genes. The occurrence of multiple unique T5orf172 endonuclease homologs within ICP1’s genome is consistent with the cycles of degradation and lateral expansion that are thought to drive HEG evolution (48), making it likely that these endonucleases are HEGs or HEG derived.

Previous research on HEGs in yeast found that individual HEGs can be associated with both sequence convergence and divergence above background rates (49). This is also seen in ICP1 where several core modules are interrupted or flanked by an endonuclease gene, while divergent regions in ICP1 genomes often contain endonucleases in one allele but not another. One such region of divergence occurs with the T5orf172 domain coding gene *tcgC*, which co-occurs with *helB* but not *helA* (Figure 4d; details in Supplemental Table 3). Such an arrangement suggests that TcgC might target *helA* or a neighboring sequence for homing. By contrast, *tcgA* and *tcgB* flank the capsid morphogenesis module that is conserved in all ICP1 isolates (Figure 2). As in other systems, some of the putative ICP1 HEGs disrupt genes existing in putative introns or inteins, peptide sequences that splice after translation. ICP1’s sole LAGLIDADG endonuclease is encoded by an intein within the highly conserved anaerobic nucleotide reductase gene *nrdA* (Figure 4c), a particularly common site for intragenic HEGs in other phages (50). An intron-encoded HNH domain coding gene, *hdgA*, divides the tape measure gene of nearly every sequenced ICP1 isolate. A single exception occurs in the oldest sequenced isolate, 1992_Ind_M4, which encodes two separate genes, *hdgB* and *hdgC*, in the same location (Figure 4e). This odd occurrence of a sole variant in the oldest isolate might reflect the eventual fixation of certain HEG alleles in the larger ICP1 population, providing an explanation for why certain HEGs appear to be associated with high conservation rather than diversity.

### 3.3. Relationship to Other Phages

As more phage genomes have become available, we have reexamined ICP1’s place in the overall tree of known prokaryotic viruses. Phylogenetic analysis reveals ICP1 isolates are much more closely related to each other than to the next-closest phage genomes on the tree, which are also *Myoviridae* that infect *Vibrio* and other Gammaproteobacteria (Supplemental Figure 1). To confirm these relationships, we performed a ViPTree analysis (51) using our entire collection of 67 ICP1 isolates and a curated list of other phage genomes (52–55) (Supplemental Table 4), which includes T4 as an outgroup. The resulting phylogeny (Figure 5a) shows even more clearly that variation among the ICP1 lineage is much lower as compared to the other putatively related phages. As this phylogenetic method uses the similarity of (at least partially) conserved ORFs to determine distance, the primary factor driving the differences seen is due to the overall genetic complement. We therefore also examined the presence/absence of several key functional elements from ICP1 in the related phages (Figure 5a), which clearly shows a large number of component similarities between the two groups but also numerous differences as would be expected from their relative positions in the phylogeny. Comparison of the capsid operon from ICP1 and a related phage, PWH3a-P1, highlights increased modularity between these groups relative to the ICP1 isolates. For example, only parts of the capsid operon, such as the portal, decoration, and coat, are similar while the proteases are more divergent (Figure 5b). Meanwhile, another phage, 11895-B1, encodes a similar terminase to ICP1 that is preceded by a dissimilar T5orf172 domain coding gene that we expect leads to splicing resulting in a fusion of the two terminase genes (Figure 5d). Several related phages share homologs to the antiholin ArrA, fewer to the holin TeaA, and only three have both. Notably, ICP1 and some related phages have an lncRNA upstream of their lysis genes. Although the sequence of the lncRNA can be highly divergent between phages, the presence of a lncRNA appears to be a conserved feature of this locus (Figure 5c). A less-conserved feature across ICP1 isolates, the CRISPR-Cas system, was found in another vibriophage isolated off the coast of Massachusetts (55) (Figure 5e). Together, these data suggest that although ICP1 isolates belong to a unique lineage, some of their seemingly unique features may be more common in other vibriophages than previously appreciated.

## 4. INTERACTIONS BETWEEN ICP1 AND *VIBRIO CHOLERAE*: DEFENSE AND COUNTERDEFENSE

Phages and their bacterial hosts are in a constant coevolutionary arms race where hosts evolve mechanisms to subvert phage predation and phages evolve counterstrategies to overcome these defenses. Like all bacteria faced with constant assault by phages, *V. cholerae* has evolved extracellular and intracellular defenses to interfere with lytic phage infection. Bioinformatic approaches have fueled the recent discovery of a large diversity of phage defense systems, many of which have been reported in *V. cholerae* (56–60). However, it remains to be determined if most of these systems are present and active in strains that ICP1 encounters in nature. For example, a CRISPR-Cas adaptive immune system is present in classical *V. cholerae* strains but absent in El Tor strains (19). Because classical strains are viewed as having gone extinct as they have not been isolated in recent decades, this suggests that ICP1 does not typically face CRISPR-Cas-mediated restriction as it infects contemporary El Tor strains. Further, given the persistence of ICP1 in nature, it is likely that ICP1 encodes counterdefenses against systems common to many *V. cholerae* strains, such as retrons (59) and cyclic-oligonucleotide-based antiphage signaling systems (60), potentially masking the antiphage activity of these systems in *V. cholerae*. Here, we focus our discussion on the defense systems that have been shown experimentally to limit ICP1 predation.

### 4.1. *Vibrio cholerae*–Encoded Defenses

**Figure 6:**
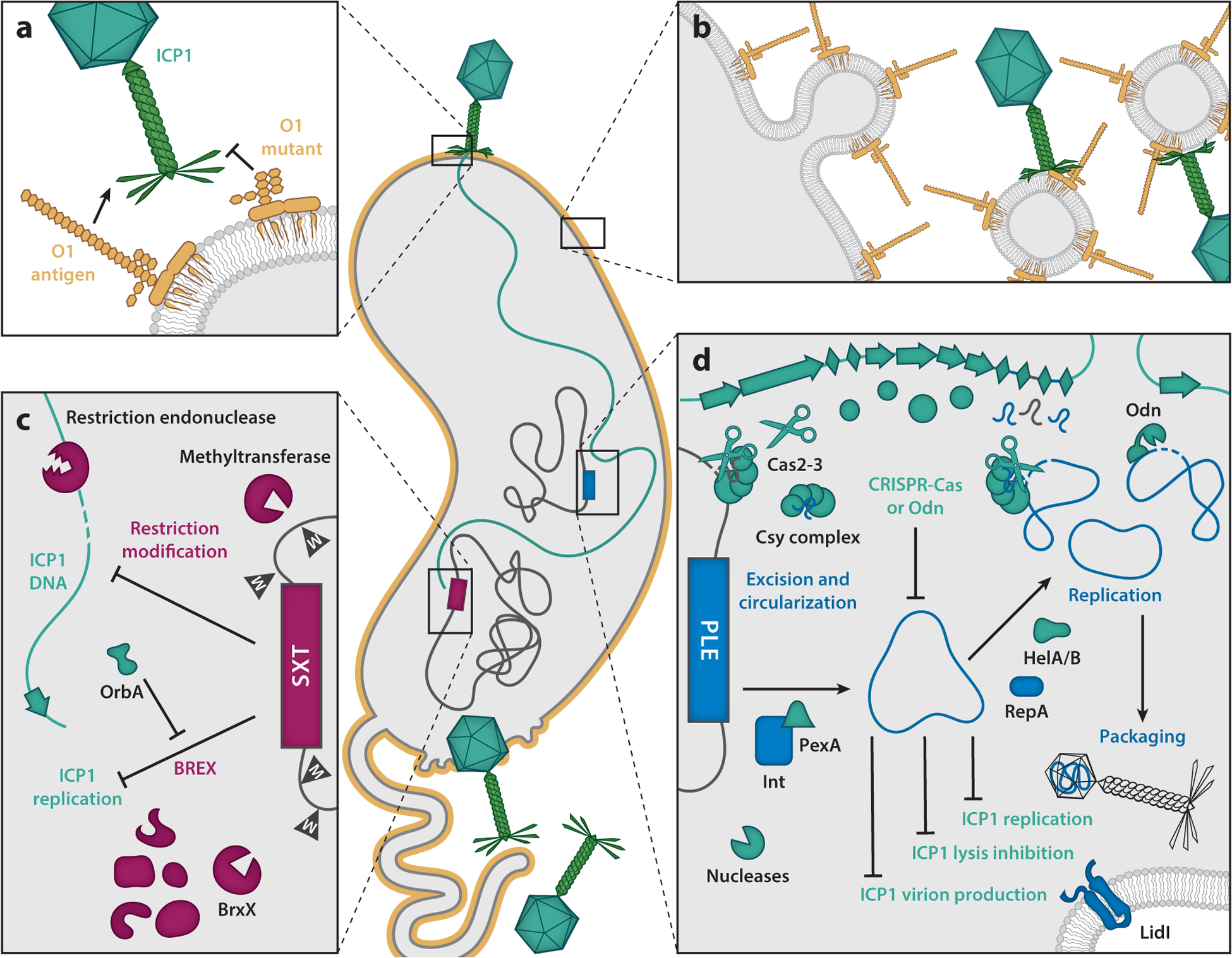
*Vibrio cholerae* defense and International Centre for Diarrheal Disease Research, Bangladesh cholera phage 1 (ICP1) counterdefense strategies. Shown is a schematic of bacterial defense strategies and phage counterdefense strategies during ICP1 (*teal*) infection of *V. cholerae* (*gray*). *V. cholerae* protects itself from ICP1 infection by (*a*) mutating the phage receptor, O1 antigen, or (*b*) releasing O1-studded outer membrane vesicle decoys (62). Alternatively, *V. cholerae* can target ICP1 DNA using (*c*) integrative and conjugative elements (ICEs) of the SXT family (variants of ICEs known for providing resistance to sulfamethoxazole and trimethoprim) (*maroon*) carried on the large chromosome. SXT ICE variants encode restriction-modification (RM) or bacteriophage exclusion (BREX) systems, the latter of which is blocked by ICP1’s OrbA (15). Finally, protection of the *V. cholerae* population is achieved by (*d*) phage-inducible chromosomal island–like element (PLE)–mediated restriction of ICP1 progeny production (23). The PLE (*blue*) resides in the small chromosome, and in response to ICP1 infection, PLE excises and interferes with lysis inhibition while blocking phage replication and virion production by hijacking ICP1-encoded proteins for its own transduction (20, 41, 65). As a counterdefense against PLE, ICP1 has evolved to degrade PLE DNA by encoding either a CRISPR-Cas system (11) that deploys the nuclease Cas2-3 or the origin-specific nuclease Odn (42). Counterdefenses against PLE allow ICP1 to replicate and complete its lytic life cycle.

Attachment to the cell surface via the O1 antigen is the first step of ICP1 infection; therefore, it is advantageous for *V. cholerae* to alter this LPS molecule. Although O1 mutations arise readily in the lab and are sufficient to block the attachment of ICP1 (61) (Figure 6a), the requirement for O1 during human infection imposes mutational constraints on the receptor. As an alternative, *V. cholerae* may protect itself from phages by shedding outer membrane vesicles (OMVs) as decoys to spare intact cells from infection (62) (Figure 6b). Upon ingestion, *V. cholerae* releases OMVs, which can enhance its virulence in the human host (63). It is unknown, however, if the doses of OMVs shown to protect against ICP1 in vitro (62) are relevant in vivo as the amount of naturally shed OMVs during human infection has yet to be quantified.

**OMV:**

outer membrane vesicle

Once a phage has injected its DNA into a host cell, it likely encounters a diverse set of defenses aimed at halting phage replication. Restriction-modification (RM) systems are a widespread defense targeting phage DNA and a common obstacle for ICP1. RM systems in *V. cholerae* can be carried on integrative and conjugative elements (ICEs) belonging to the SXT family (Figure 6c). SXT ICEs are well studied for their role in conferring resistance to antibiotics including sulfamethoxazole and trimethoprim (64), antibiotics that had once been useful for treating cholera. SXT ICEs encode conserved genes required for intracellular maintenance and horizontal transfer but also carry variable genes in hotspot regions that confer element-specific phenotypes. Notably, RM systems cluster in hotspot 5 of some SXT ICEs, which contributed to the recent discovery of SXT ICEs mediating antiphage defense (15). In all SXT ICEs found in toxigenic O1 *V. cholerae* strains, hotspot 5 encodes phage resistance genes—these include several RM systems in ICE*Vch*Ind6 and distinct BREX systems in ICE*Vch*Ind5 and ICE*Vch*Ban9. These three SXT ICEs, which are the globally dominant variants in toxigenic O1 *V. cholerae*, restrict ICP1 as well as other phages (15). These are sequence-specific restriction systems wherein phage DNA is recognized as foreign by its lack of methylation, and, as such, ICP1 could overcome restriction by introducing the appropriate epigenetic modifications. However, SXT ICE heterogeneity in the *V. cholerae* population introduces a variety of distinct modification requirements the phage could encounter and thus serves as an important factor limiting ICP1.

**RM:**

restriction-modification

**ICE:**

integrative and conjugative element

**SXT:**

variant of ICE known for providing resistance to sulfamethoxazole and trimethoprim

In contrast to SXT ICE-mediated phage defenses, which protect the cell from diverse phages (15), phage-inducible chromosomal island–like elements (PLEs) provide *V. cholerae* with specific defense against ICP1. PLEs completely abolish ICP1 progeny production and parasitize phage resources to promote their own spread (Figure 6d). In this way, PLEs can be viewed both as defense systems for *V. cholerae* and also as phage satellites that exploit ICP1 for their own mobilization. PLEs are found integrated in the *V. cholerae* genome in uninfected cells but excise in response to ICP1 infection (11, 65), replicate (21, 23), and steal structural proteins from ICP1 to package their own genome into modified transducing particles (20). The specificity for PLEs’ response to ICP1 is driven by an ICP1-encoded protein called PexA, which serves as the trigger for PLE excision by interacting with the PLE-encoded integrase (Int) (65). PexA is found in all ICP1 isolates sequenced to date but is not found in other phages, explaining why PLEs do not respond to other vibriophages (23, 65). Although the PLE(+) *V. cholerae* cell dies as a result of ICP1 infection, by blocking the production of ICP1 progeny, PLEs provide protection for the *V. cholerae* population as a whole. At least five genetically distinct PLEs have been circulating in epidemic *V. cholerae* since as far back as 1949 (23). All characterized PLEs have anti-ICP1 activity (23), yet each has different combinations of alleles that contribute to ICP1 inhibition, suggesting that PLEs are under selective pressure to diversify and yet maintain their modus operandi.

**PLE:**

phage-inducible chromosomal island–like element

The basis of PLE-mediated inhibition of ICP1 is an active area of study, and several recent discoveries have revealed distinct mechanisms that PLEs use to disrupt ICP1’s life cycle. PLEs interfere with ICP1 genome replication and use their own replication initiation factor (RepA) and redirect ICP1’s replication machinery to outcompete ICP1 replication and produce eight-fold more replicated genomes than ICP1 (21). Interestingly, PLEs can exploit either of ICP1’s helicases, HelA or HelB, which vary between ICP1 isolates (Figure 4d), for replication and are themselves dispensable for ICP1 replication (14). Beyond these helicases, it is not known exactly which components of ICP1’s replisome are hijacked by PLEs, nor is it known how that hijacking is achieved. Simultaneously, PLEs interfere with ICP1’s transition to rolling circle replication, stalling the production of concatemers needed for packaging (21). Now, the abundant, presumably concatemerized PLE genomes can be packaged into modified virions, which have a smaller capsid (20). Preliminary data suggest that PLE virions are composed of primarily hijacked structural proteins encoded by ICP1. By hijacking and modifying ICP1’s capsid, PLEs can efficiently package their smaller ~18-kb genome but exclude packaging of ICP1’s ~126-kb genome. The mechanisms that control PLE-mediated virion remodeling and the potential preference in packaging the PLE genome over ICP1’s genome are still under investigation. At one of the final stages of infection, PLEs use LidI to disrupt lysis inhibition, interfering with ICP1’s strategy to increase the number of progeny before lysis (41). PLEs’ strategy of targeting essential stages in ICP1’s life cycle limits the phage’s ability to escape through mutation and instead leads to the evolution of anti-PLE mechanisms. PLE-mediated inhibition of ICP1 serves as a tractable model system to deepen the mechanistic understanding of how parasites of viruses can be co-opted for host defense, which has occurred multiple independent times across diverse virus-host systems (66–70).

### 4.2. ICP1-Encoded Counterdefenses

*V. cholerae*’s anti-ICP1 defenses, namely SXT ICEs and PLEs, have been met by reciprocal adaptations in ICP1 that ensure its continued success in nature. ICP1 can escape restriction by SXT ICEs by epigenetic modification wherein a small number of infecting phage genomes evade restriction and are subsequently modified by the cognate methylase (15). These modified phages then spread unhindered in the presence of cognate SXT ICEs. ICP1 isolates also encode an anti-BREX protein, OrbA, to escape ICE*Vch*Ind5-mediated inhibition (15) (Figure 6c). While the only other known anti-BREX protein, encoded by T7 phage, is a DNA mimic (71), OrbA appears to function through a unique mechanism that remains to be elucidated. Although *orbA* is considered a core gene common to all ICP1 isolates, some isolates possess a deletion encompassing the *orbA* promoter that leaves them sensitive to restriction by ICE*Vch*Ind5 (15).

PLEs deploy multiple mechanisms to inhibit ICP1, and ICP1 has in turn evolved multiple anti-PLE mechanisms. Two anti-PLE mechanisms have been described to date, both of which harness a nuclease to target the PLE genome for degradation (Figure 6d). Of the ICP1 isolates sequenced to date, 40% use Odn (Figure 4b), an endonuclease that targets PLE’s *ori* by mimicking the PLE-encoded replication initiation factor, RepA (42). Interestingly not all PLEs use the same RepA protein and cognate *ori* sequence (which together comprise the replication module), and that diversity protects some PLEs from Odn-mediated attack, supporting the hypothesis that PLE diversification is driven, at least in part, by ICP1’s anti-PLE mechanisms. Given that certain PLEs are insensitive to Odn, the remaining 60% of ICP1 isolates encode CRISPR-Cas in place of Odn (11) (Figures 4b and 6d) (42).

### 4.3. ICP1’s CRISPR-Cas System

ICP1 was the first phage shown to encode a fully functional CRISPR-Cas system (11). This Type I-F system, like others, uses Cas1 to cleave and integrate spacers from foreign DNA, which are transcribed into precursor CRISPR RNAs and processed by Csy4, and complexed with the effector complex comprising Csy1, Csy2, and Csy3 (72). Binding of a matching target sequence (protospacer) with the appropriate protospacer adjacent motif (PAM) and recruitment of the transacting nuclease Cas2-3 leads to sequence-specific degradation (11, 13). To date, the dozens of cataloged distinct spacers in CRISPR(+) ICP1 isolates with identified targets are specific to sequences in PLEs (13) (Figure 6d). Rare spacers without known matches or matches to *V. cholerae*’s chromosome have been identified (11, 13), suggesting additional PLEs remain to be discovered or perhaps that ICP1’s CRISPR-Cas system could be used against non-PLE targets. Notably, ICP1’s CRISPR-Cas system is not similar to the Type I-E system carried by classical *V. cholerae* strains, and a cellular origin for ICP1’s CRISPR-Cas system has yet to be identified (11). The Cas1 proteins in ICP1 and vibriophage 1.161 have diverged significantly from cellular Cas1 proteins, suggesting a relatively ancient acquisition event and/or a fast rate of evolution following viral capture (73). The CRISPR-Cas system in the vibriophage 1.161 (Figure 5e) remains to be characterized, so it is unclear if it is functional and what its targets are. However, we speculate that although PLEs have yet to be identified outside of *V. cholerae*, given its similarity to ICP1, vibriophage 1.161 likely uses its CRISPR-Cas system against its own PLE-like parasite resident in its host, *Vibrio lentus*.

**PAM:**

protospacer adjacent motif

ICP1 and vibriophage 1.161 are not the only phages encoding CRISPR-Cas systems. Recent expanded metagenomic sequencing efforts show that these co-opted systems are a more common feature of phage genomes than previously recognized (73). One reason they were not initially documented is that these systems often occur only in fragments, missing one or more components and, in some cases, consisting of a single gene or solitary repeat unit, making them difficult to identify (73, 74). Unlike the complete set of genes in ICP1, and putatively in vibriophage 1.161, which can act autonomously during infection, these fragmented systems are hypothesized to partially rely on host-encoded CRISPR machinery. In this case, the phage likely hijacks the host’s proteins to acquire spacers, which it can then deploy to target competing phages’ DNA for degradation during coinfection (74). The most reduced phage-encoded system studied thus far comes from a group of giant phages that encode only a small array (an average of five spacers) and a single, multifunctional Cas protein that is 50% the size of the well-studied Cas9 nuclease (75). Similarly, ICP1’s system encodes smaller proteins than those in other Type I-F systems such as the well-studied system found in *Pseudomonas aeruginosa*. For example, ICP1’s Csy1 and Csy2 proteins are about 40% and 75% the size, respectively, of *P. aeruginosa*’s. The compact nature of most viral genomes requires that these CRISPR-Cas systems are streamlined, making them especially appealing for biotechnology applications (75). Not only do minimal CRISPR-Cas systems from phages make for simple constructs to implement in other species for genome editing, but also the diverse PAMs they target (11, 13, 75) may afford expanded opportunities for genetic engineering. Additionally, phage-encoded CRISPR-Cas systems are a useful model for deciphering how viruses co-opt and repurpose components of cellular defenses.

## 5. SUMMARY AND FUTURE DIRECTIONS

Since its discovery, substantial progress has been made in characterizing ICP1, and these efforts have uncovered some fascinating features of this lytic phage, yet much remains unknown. In-depth analyses of its life cycle under laboratory conditions revealed an exquisitely coordinated pattern of gene expression and facilitated functional predictions of many of ICP1’s gene products. However, the vast majority of ICP1’s gene products remain functionally mysterious, lacking homology to other proteins or domains. While regulatory mechanisms underpinning ICP1’s gene expression pattern have yet to be determined, characterization of ICP1’s lncRNA as well as other genes without predicted functions should be addressed in future research. It is curious that only a few vibriophages, the dominant of which is ICP1, are recovered from cholera patient samples (10), suggesting ICP1 may be more tolerant to passage through the gastrointestinal tract or uniquely poised to amplify in this environment. Importantly, to address ICP1’s effect on limiting disease, more data are needed to reconcile the discrepancy between a protective role for ICP1 against cholera seen in animal models (6, 77, 78) and the continued prevalence of both ICP1 and toxigenic *V. cholerae* in patient stool. Perhaps other factors to be considered are metabolic changes in *V. cholerae* within the human host that limit ICP1’s capacity to infect or lyse these cells. Indeed, ICP1’s life cycle in the intestinal environment has yet to be explored.

Interestingly, genomic analysis of this persistent predator revealed surprisingly high levels of sequence conservation between ICP1 isolates, even among those isolated decades apart. The modular nature of the genome and high density of putative HEGs suggest ICP1 phages evolve largely via recombination, which contributes to how these isolates cluster separately from other phages. As some of ICP1’s characteristics are shared by other phages, it will be interesting to see, as more phages are isolated and sequenced, if ICP1 remains a unique lineage or if there are more closely related phages than those we have identified thus far. Deeper analysis of variations between ICP1 genomes has revealed instances of allele swaps, several of which can be linked to counterdefense against particular variants of resident antiphage elements in *V. cholerae*. In the future, analysis of novel ICP1 and *V. cholerae* isolates from aquatic reservoirs and cholera patient samples will likely continue to reveal fascinating biology that contributes to the finely tuned, perpetual balance achieved in this phage-host system.

## Supplementary Material

S1 figure

Supplemental Tables 1-5
