# Supplementary material for: Bacteriophage ICP1: A Persistent Predator of Vibrio cholerae
Source: Annu Rev Virol. Author manuscript; Available in PMC 2022 Apr 26. (PMC9040626; doi:10.1146/annurev-virology-091919-072020)
Supplement: S1 figure [file NIHMS1792863-supplement-S1_figure.pdf]

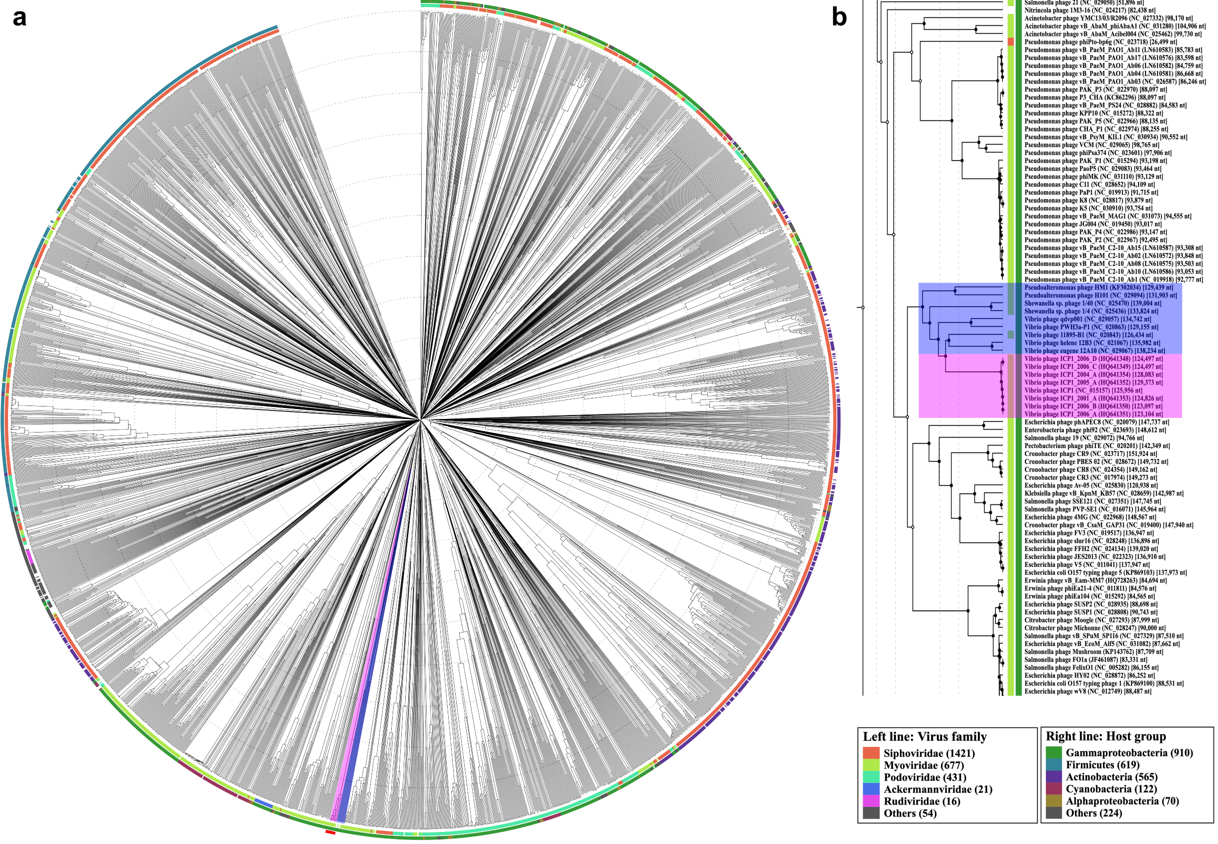

Supplemental Material: Annu. Rev. Virol. 2021. 8:285-304
https://doi.org/10.1146/annurev-virology-091919-072020
Bacteriophage ICP1: A Persistent Predator of Vibrio cholerae
Boyd, Angermeyer, Hays, Barth, Patel, and Seed
